# A study protocol for facility assessment and follow-up evaluations of the barriers to access, availability, utilization and readiness of contraception, abortion and postabortion services in Zika affected areas

**DOI:** 10.1186/s12978-017-0283-8

**Published:** 2017-02-02

**Authors:** Moazzam Ali, Rachel Folz, Kelsey Miller, Brooke Ronald Johnson, James Kiarie

**Affiliations:** Department of Reproductive Health and Research, World Health Organization, Avenue Appia 20, Geneva 27, CH-1211 Switzerland

**Keywords:** Zika virus, Health facilities, Rapid assessment, Contraception, Abortion care, Post-abortion care, Latin America

## Abstract

**Background:**

The Zika virus epidemic in Latin America has elicited official recommendations for women to delay or avoid pregnancy in affected countries, which has increased demand for family planning services. It is likely, however, that health facilities in areas where the population is most vulnerable to the disease lack the capacity to respond to the increased demand for family planning services. Our objectives are to perform facilities assessment and understand client perceptions in areas affected by Zika virus, and to track changes in these parameters over time.

**Methods/design:**

We will collaborate with local health authorities to map facilities that have the capacity to provide services in contraception and safe abortion, including induced abortion to the full extent of the law and post-abortion care for treatment of complications from unsafe abortion and post-abortion contraception. We then will carry out a survey of facilities to assess the availability of services and their readiness to provide contraception and safe abortion care. All facilities will be assessed for baseline readiness and availability of services, and a random subsample of surveyed facilities will be reassessed in second and third rounds of surveys. Focus group interviews with clients will be conducted as part of the facilities surveys in order to gain an understanding of the community’s knowledge, needs and perceived barriers to healthcare in the context of the Zika virus epidemic.

**Discussion:**

The findings of this study will aid the response to Zika virus ranging from the identification of healthcare facilities that can be potentially strengthened, to the formulation of interventions to reduce barriers and improve readiness of facilities to provide contraception and safe abortion services. Lessons learned from this study will help to build and strengthen health systems that are more prepared to consistently providing reproductive healthcare services in the context of health emergencies.

## Plain English Summary

In the wake of the Zika virus epidemic in Latin America, governments have suggested that women who want to have children should wait before becoming pregnant to reduce their risk of having a baby with microcephaly or other abnormalities caused by Zika virus. If women are to follow these recommendations, they will need family planning services such as contraception and access to safe abortion. It is likely, however, that healthcare facilities in areas where women are most vulnerable to Zika virus are not prepared for the increased demand for family planning services. Our objective is to assess the status of health facilities in areas affected by Zika virus.

Over the course of project timeline we will perform three rounds of surveys of health facilities in affected areas to determine what contraception, abortion and post-abortion care services they provide and their readiness to deliver these services. We will also conduct focus group interviews with people who are served by the facilities to gain an understanding of the community’s knowledge, needs and perceived barriers to receiving healthcare in the context of Zika virus.

This study will help us identify which facilities are prepared to respond to the Zika virus epidemic, and which need to be strengthened. It will also help identify general gaps in services provided and barriers that women, men, and adolescents must overcome to get the care that they need. Lessons learned from this study will help to build health systems that are more prepared to consistently provide reproductive healthcare services in the context of emergencies.

## Background

Zika virus (ZIKV) is a mosquito borne virus that has been shown to cause microcephaly and other severe brain anomalies in newborns when the mother is infected prenatally [1, 2]. ZIKV during pregnancy can result in many adverse events such as fetal deaths or newborns with congenital abnormalities including microcephaly and other neural irregularities [3, 4]. In light of this, the WHO declared the ZIKV outbreak to be a Public Health Emergency of International Concern (PHEIC) in February 2016. At this time, the WHO recommended that risk of infection to women of reproductive age should be minimized in affected countries and that the capacity for surveillance of the disease should be strengthened [5].

Because of the possible adverse outcomes in pregnancy caused by ZIKV, many women and adolescents want to postpone or avoid pregnancies. Some health ministers in Latin American countries have officially recommended that women postpone or avoid pregnancy, and interim recommendations from the WHO to prevent ZIKV infection in affected areas include the provision of emergency contraceptive services, counseling to women that have had unprotected sex, and access to resources for delaying pregnancy [6]. Regarding the risk of sexual transmission of ZIKV, the official recommendations encourage practicing safe sex with condoms to avoid continued spread of the disease [6]. We have already seen, and further anticipate, a significant increase in demand for both contraception and safe abortion services [7].

However, we suspect that facilities in the most affected regions lack the capacity to respond to the increased demand for family planning. There may be inadequate infrastructure and delivery systems, inadequate commodities and supplies (including emergency contraception, long-acting reversible contraception, condoms, electric and/or manual and vacuum aspiration (MVA), and mifepristone and misoprostol) and/or a lack of trained personnel to provide quality care to meet the needs of the population. Due to the suspected lack of provision of adequate contraceptive and safe abortion services, as well as severely restrictive abortion laws, we fear that many women will be forced to choose clandestine and unsafe abortion methods. This highlights the importance of readiness to provide post-abortion care in these areas, even though induced abortion may be illegal in many circumstances.

While the rate of modern contraception use in Latin America and the Caribbean has increased and the fertility rate has declined over recent decades [8, 9, 10] there are still about 23 million women in the region who wish to avoid pregnancy but are not using contraception [11] and 10% of all maternal deaths in the region are due to unsafe abortion [12]. Additionally continuation rates for contraception are especially low in Latin America [9]. All of these factors likely contribute to the fact that the Latin America and Caribbean region has the highest proportion of unintended pregnancies in the world [13]. Additionally 97% of women of reproductive age in Latin America live in areas where abortion is restricted or banned altogether [14]. Within our study region, abortion is legal only in cases of rape/incest, anencephalic fetus, or to save a woman’s life in Brazil, and legal to save a woman’s life, preserve her physical and/or mental health, in cases of rape/incest, or because of fetal impairment in Colombia [15]. While it is difficult for any woman to access to safe abortion in Brazil, this is disproportionately true for women of lower socio-economic status, and safe abortion access in Brazil is one example of extreme inequity in healthcare [10]. Access to family planning services are likely inequitable in all of our study countries, which exacerbates the challenges presented by ZIKV.

As is commonly seen in public health emergencies, disease burden is often highest for populations of lower socioeconomic status due to a disproportionate lack of access to quality health care. As was true with Ebola, this is also likely the case for Zika virus [16, 17]. Poor populations often have a greater risk of infection due to poor sanitation, lack of clean water, and overcrowding. Additionally, higher-quality public and private health care tend to be more readily available and accessible to people of higher socioeconomic status, thus contributing to the perpetual cycle of poor health and inequality, which is exacerbated during epidemics or disasters [18]. In the Ebola outbreak, many weak health systems quickly collapsed in wake of the disease and failed to provide basic healthcare to vulnerable populations. Though reproductive health was crucial in the context of Ebola, which is suspected to be sexually transmitted, health systems were not able to provide adequate services for women and children, especially in the context of reproductive health [19, 20]. ZIKV now puts a spotlight on the state of reproductive health services in Latin America. In order to apply the lessons learned from the Ebola outbreak and prevent the foreseen exacerbation of existing gender inequalities and social injustices [21], it will be essential to focus on ensuring equitable quality care in reproductive health if we hope to adequately serve the populations that are most vulnerable to Zika virus.

### Study aims and objectives

It is essential to gather data on the impact that Zika is having in communities and on healthcare systems, and little of this type of work has been done to date. We hope to contribute to filling this evidence gap by conducting facility assessment of contraception and safe abortion/post-abortion care services available in ZIKV-affected areas in Brazil, Honduras and Columbia. Through this facility assessment, we will gather information on the types of contraception and abortion/post-abortion care services available in local health centers, and assess the local health facilities’ readiness in addressing concerns and providing quality care to clients of all socioeconomic statuses. In assessment of abortion services, keeping in mind that in many settings abortion is either highly restrictive or not permitted, we will follow the National policy and guidelines and will assess only the services allowed by law. We will also assess the client’s perspective of services and barriers to utilization of services.

In the short-term we hope to begin to conduct a much needed assessment of the state of facilities in ZIKV-affected areas to provide the Ministries of Health with information that will help them more equitably serve the general population. The long-term goals that we hope to achieve are to strengthen the health system’s response to the sexual and reproductive health needs of the communities, and advocate for equitable access to essential reproductive health services.

The specific objectives of this study are the following:

1. to create a map of all health facilities with the capacity to provide contraceptive and abortion/post-abortion care in the ZIKV-affected areas in Brazil, Honduras and Columbia.
2. to conduct assessment of facilities selected at subnational level that are most affected by ZIKV, regarding availability and readiness to provide services in contraception and abortion care, including treatment of complications from unsafe abortion and provision of post abortion contraception.
3. to assess client perceptions of services and barriers to service utilization in selected ZIKV-affected areas.
4. to assess changes in availability and readiness of facilities to provide services in contraception, abortion and post abortion care, and changes in client’s perspectives after Ministries of Health are provided with baseline data and given time to make changes to health systems.

## Methods/design

This study will employ a quantitative survey and qualitative stocktaking to assess health facilities and client perceptions. The study will be carried out in collaboration with the Ministries of Health in countries, with WHO regional and country offices and with local academic institutions.

### Mapping of facilities in ZIKV-affected areas

The principal investigator, with the help of the Ministries of Health, will identify and map all facilities in identified ZIKV-affected areas in Brazil, Honduras and Columbia with the potential to provide services in contraception and safe abortion including treatment of complications and provision of post abortion contraception care. The affected areas will be identified jointly by the Ministry of health and WHO country office.

### Health facilities surveys

The principal investigator will train the local project team on the study purpose, design, quality assurance, reporting mechanisms and data collection tools. All facilities identified in the mapping activity will then be visited, data collection will be done and assessed. This data will generate evidence on availability of services and health facilities’ readiness to deliver services in contraceptive and abortion care and treatment of complications and provision of post abortion contraception care.

A cross-sectional panel survey design will be used. In the first round of surveys, we plan to collect data from all identified facilities’ records in the ZIKV-affected areas with the potential to provide services in contraception, safe abortion and post-abortion care. The survey team will interview the person most knowledgeable (senior health care provider and/or administrator) about contraceptive and abortion care and treatment of complications and provision of post abortion contraception care in the facility.

Baseline data will be followed with two more data points at 6 months and 12 months after the ministries of health have received detailed feedback on the first data report. It is expected that based on the findings the Ministries of health will take corrective measures to strengthen facilities. In the second and third phases of data collection, we plan to use a random subsample of the facilities from the original sampling framework to assess trends in service availability and readiness over time. We anticipate challenges in gathering data about availability and readiness to perform safe abortions, but in these cases we plan to still assess readiness and availability of services to provide post-abortion care.

The following aspects of facilities will be assessed using validated tools:

1. supplies and equipment for contraception (adapted from the Service Availability and Readiness Assessment (SARA) guide laid out by the WHO) [22].
2. safe abortion care and post abortion contraception (adapted from the WHO’s safe abortion assessment tool) [23].
3. Focus group discussion, adapted from Columbine M, Busza J, Cleland J, Campbell O. Social science methods for research on sexual and reproductive health. Geneva: World Health Organization; 2012 [24].

Here “availability” refers to facility and health worker physical locations, numbers, and distribution per capita. “Readiness” refers to the capacity to deliver services (including basic amenities, equipment and supplies, diagnostics, essential medicines and commodities, and competencies of healthcare providers).

### Client’s perspective

A qualitative descriptive study with focus group interviews will be used to provide an in-depth understanding of clients’ perspectives in ZIKV-affected areas. Using the WHO’s guideline for focus group interviews, a guide for focus group interview facilitators will be prepared by the PIs and FGD experts by identifying the main objectives of the meetings and developing key questions.

Information gathered will include:

1. knowledge about Zika virus (such as cause, signs and symptoms)
2. care seeking and perception of risk about being infected by Zika virus
3. knowledge about microcephaly and other neurological defects related to Zika virus
4. needs related to sexual reproductive health services (including contraception and abortion care)
5. barriers to accessing reproductive health services

Participants will be recruited for the focus group interviews based on criteria such as experiences on access to particular health services, socio-demographic characteristics and comfort with other fellow participants in the group. The principal investigator and project team along with local health authorities will visit the local communities to identify potential participants. We plan to recruit participants who are women and men from all socioeconomic backgrounds in the area, of reproductive age, who have sought or tried to receive health services from local health facilities. Two to three focus groups will be interviewed in selected geographic area (state or a district). We plan to follow these focus groups over time, to understand the change in risk-perception, needs and concerns regarding the Zika epidemic. Focus group discussions will be conducted at the same time and in the same place as facilities assessments to understand how both factors change together over time.

### Data management and analysis

A service readiness assessment will be conducted to fill critical data gaps in service delivery and data quality using the WHO/USAID Service Availability and Readiness Assessment (SARA) tool, as a primary data source for the analytical review. The SARA provides key information on the state of the health system in terms of service availability, as well as the readiness of the facilities to provide an adequate level of service. Monitoring facility-level performance provides information on whether health services are present and are being provided at the expected level, and gives an indication of how investments in the formal health sector are resulting in changes at the level of service delivery. This affects utilization of services and ultimately impacts population-level outcome measures.

Providing an enabling working environment is a requirement for an effective and functional health care delivery system. Such enabling environment includes the physical infrastructure and the availability of basic requirements for delivering quality services. The assessment included a review of health facility records in family planning and contraception, to assess the availability, type and range of commodities offered.

Analysis of readiness to provide family planning services was assess based on the presence of the six tracer items i.e. guidelines for family planning, staff trained in family planning in the past 2 years, blood pressure apparatus, combined oral contraceptive pills, injectable contraceptives and male condoms.

Quantitative data from the mapping and facility assessment survey will be entered into an electronic database using SPSS and Epidata 3.1. The data will be from the facility’s situation analysis i.e. interview with key staff and facility records. Descriptive analysis will include computing the frequencies and proportions of categorical variables while mean and other measures of central tendency will be computed to describe continuous variables.

Qualitative data will be analyzed using qualitative analytical tools such as NVIVO. It will be based on information collected through focus groups with clients in the ZIKV-affected sites in the catchment areas of health facilities. Qualitative content analysis will be used to analyze the focus group interview data. All interviews will be transcribed verbatim and then analyzed following the general approach of content analysis. The coding process strategy will focus the data analysis on information that unexpectedly emerges from empirical evidence. Two researchers will analyze the data and will be guided by general questions, specific questions, and comparisons. The coding method and progress will be systematically discussed between the researchers and the project coordinator. The results will be translated into English. Data will not include any identifying information of any participant, and instead ID numbers will be used for identification.

The HQ team based in Geneva, in conjunction with PI and project team, will be responsible for the data analysis. The analysis process will begin while data collection is still on-going in order to assess progress and determine any data collection problems and/or patterns.

### Quality assurance

The investigator and project team will be trained on the study purpose, design, quality assurance, reporting mechanisms and data collection tools. Data collectors will also be trained to use the study questionnaire/tool for data collection from the facilities and entering the survey data. Experts will conduct the focus group discussion. Surveyors will be trained on data collection, transmission, verification, storage and primary analysis to assess errors.

All data will be double-entered into existing software used by two separate data operators. Measures will be taken to ensure the quality of collected data. All the forms will be checked on a daily basis for completeness, logical errors, and unclear or irrelevant responses. The principal investigator (PI) and project team will make monitoring visits to ensure the quality of data and adherence to the study protocol. If needed, refresher training will be arranged to enhance understanding of data collection tools.

### Scientific and ethical review

Scientific review and approval of the proposal has already been obtained from Research Projects Review Panel, the external review body of the Department of Reproductive Health, and Research (WHO/RHR) including the UNDP/UNFPA/WHO/World Bank Special Programme of Research, Development, and Research Training in Human Reproduction (HRP). Ethical approval has also been obtained from WHO Research Ethics Review Committee.

### Project deliverables

Key outputs from this study will be as follow:

1. a map of all facilities providing contraception and abortion/post-abortion care services in selected ZIKV-affected areas of participating countries
2. facility assessment modules on contraception and abortion/post-abortion care
3. assessment report with key findings and recommendations for improving service availability and quality of care in contraceptive and abortion care services
4. Key findings from focus group discussions report

### Timeline and tasks

We anticipate that this study will take approx. 18 months to complete. Tools will be developed and facilities will be mapped in affected areas during the first 2 months. After this, the data collection will commence and will take 3 months. This will involve data collector training with the study tool, followed by assessment of facilities and data collection in conjunction with focus group interviews in communities. Data entry and analysis will take 2 months. In the following 3 months, reports will be written, and dissemination meetings will be held with the Ministries of Health, health managers and stakeholders to report key findings and areas for improvement. After the groundwork is completed with the first round of assessment, there will be a 6-month break in the study before the second and third rounds of surveys are completed. The second round will consist of 2 months of rapid assessment and data collection of selected facilities and focus group interviews in communities. Data entry, analysis and comparisons will be done in the following month to see what changes have happened since the first round, after which there will be 1 month for report writing and dissemination meetings with the Ministries of Health, health managers and stakeholders. The last round of data collection and analysis will be done with the same 4-month breakdown as the second round.

## Discussion

The data derived from this project will provide a better understanding of the impact of ZIKV on contraception and abortion/post abortion care programs at the regional and community level to policy makers and health managers to develop and strengthen policies and services to be more responsive to community needs. These policies and services will relate to ensuring availability of adequate method mix and to ensuring accessible and affordable, women centred services in contraception and safe abortion.

This study will provide a map of facilities in ZIKV-affected areas in Brazil, Colombia and Honduras. It will also assess the readiness of sampled facilities to provide contraception, abortion (where possible) and post-abortion care services. These important data will be given to Ministries of Health with the aim of strengthening the health system to ensure good-quality care in ZIKV-affected areas.

The study also has implications on future steps towards addressing the ZIKV epidemic in Latin America. The results of this study will aid in understanding service gaps in health systems and formulating interventions to improve facilities’ readiness to provide contraception and abortion/post-abortion care services in the face of the ZIKV epidemic and beyond.

The results and insights gleaned from this study will guide future research and interventions that focus on overcoming access and utilization barriers, and may guide the development of a planning preparedness model for other countries i.e. provide a set of tools and processes, which can be applied in other settings, to ensure consistent quality reproductive healthcare in the context of health emergencies.

## Abbreviations

FGD: Focus group discussions
MVA: Manual and vacuum aspiration
PHEIC: Public health emergency of international concern
PI: Principal investigator
SARA: Service availability and readiness assessment
SPSS: Statistical package for the social sciences
WHO: World Health Organization
ZIKV: Zika virus

## References

[1] Rasmussen SA, Jamieson DJ, Honein MA, Petersen LR (2016). Zika virus and birth defects--reviewing the evidence for causality. N Engl J Med.

[2] Cauchemez S, Besnard M, Bompard P, Dub T, Guillemette-Artur P, Eyrolle-Guignot D, Salje H, Van Kerkhove MD, Abadie V, Garel C (2016). Association between Zika virus and microcephaly in French Polynesia, 2013-15: a retrospective study. Lancet.

[3] Costello A, Dua T, Duran P, Gülmezoglu M, Oladapo OT, Perea W, Pires J, Ramon-Pardo P, Rollins N, Saxena S (2016). Defining the syndrome associated with congenital Zika virus infection. Bull World Health Organ.

[4] Brasil P, Pereira JP, Raja Gabaglia C, Damasceno L, Wakimoto M, Ribeiro Nogueira RM (2016). Carvalho de Sequeira P, Machado Siqueira A, Abreu de Carvalho LM, Cotrim da Cunha D, et al. Zika Virus Infection in Pregnant Women in Rio de Janeiro. N Engl J Med.

[5] World Health Organization. WHO statement on the first meeting of the IHR Emergency Committee on Zika virus and observed increase in neurological disorders and neonatal malformations. Geneva: World Health Organization. http://www.who.int/mediacentre/news/statements/2016/1st-emergency-committee-zika/en/. Accessed 12 Jan 2017.

[6] World Health Organization (2016). Prevention of sexual transmission of Zika virus: interim guidance update.

[7] Aiken AR, Scott JG, Gomperts R, Trussell J, Worrell M, Aiken CE. Requests for Abortion in Latin America Related to Concern about Zika Virus Exposure. N Engl J Med. 2016. 10.1056/NEJMc1605389 PMC5026851 27331661

[8] Ugaz JI, Chatterji M, Gribble JN, Mitchell S (2015). Regional trends in the use of short-acting and long-acting contraception accessed through the private and public sectors. Int J Gynaecol Obstet.

[9] Ali MM, Cleland J (2005). Sexual and reproductive behaviour among single women aged 15-24 in eight Latin American countries: a comparative analysis. Soc Sci Med.

[10] Diniz SG, d’Oliveira AF, Lansky S (2012). Equity and women’s health services for contraception, abortion and childbirth in Brazil. Reprod Health Matters.

[11] Guttmacher Institute. Sexual and Reproductive Health Research Analysis: Latin America & the Caribbean. 2016. https://www.guttmacher.org/geography/latin-america-caribbean. Accessed 12 Jan 2017.

[12] Singh S, Darroch JE, Ashford LS (2014). Adding it up: the costs and benefits of investing in sexual and reproductive health.

[13] Sedgh G, Singh S, Hussain R (2014). Intended and unintended pregnancies worldwide in 2012 and recent trends. Stud Fam Plann.

[14] Sedgh G, Bearak J, Singh S, Bankole A, Popinchalk A, Ganatra B, Rossier C, Gerdts C, Tunçalp Ö, Johnson BR (2016). Abortion incidence between 1990 and 2014: global, regional, and subregional levels and trends. Lancet.

[15] United Nations (2011). Population division: world abortion policies.

[16] Menéndez C, Lucas A, Munguambe K, Langer A (2015). Ebola crisis: the unequal impact on women and children’s health. Lancet Glob Health.

[17] Garcia-Subirats I, Vargas I, Mogollón-Pérez AS, De Paepe P, da Silva MR, Unger JP, Borrell C, Vázquez ML (2014). Inequities in access to health care in different health systems: a study in municipalities of central Colombia and north-eastern Brazil. Int J Equity Health.

[18] Mutter J (2010). Disasters widen the rich-poor gap. Nature.

[19] Barden-O’Fallon J, Barry MA, Brodish P, Hazerjian J. Rapid Assessment of Ebola-Related Implications for Reproductive, Maternal, Newborn and Child Health Service Delivery and Utilization in Guinea. PLOS Currents Outbreaks. 2015. doi:10.1371/currents.outbreaks.0b0ba06009dd091bc39ddb3c6d7b0826. 10.1371/currents.outbreaks.0b0ba06009dd091bc39ddb3c6d7b0826 PMC4542265 26331094

[20] Delamou A, Hammonds RM, Caluwaerts S, Utz B, Delvaux T (2014). Ebola in Africa: beyond epidemics, reproductive health in crisis. Lancet.

[21] Roa M (2016). Zika virus outbreak: reproductive health and rights in Latin America. Lancet.

[22] World Health Organization (2015). Service availability and readiness assessment (SARA): implementation guide.

[23] World Health Organization (2012). Safe abortion: technical and policy guidance for health systems.

[24] Columbine M, Busza J, Cleland J, Campbell O (2012). Social science methods for research on sexual and reproductive health.

