# Erratum to: A study protocol for facility assessment and follow-up evaluations of the barriers to access, availability, utilization and readiness of contraception, abortion and postabortion services in Zika affected areas

**DOI:** 10.1186/s12978-017-0366-6

**Published:** 2017-08-22

**Authors:** Moazzam Ali, Rachel Folz, Kelsey Miller, Brooke Ronald Johnson, James Kiarie

**Affiliations:** Department of Reproductive Health and Research, World Health Organization, Avenue Appia 20, CH-1211 Geneva 27, Switzerland

## Erratum

The original version of this article [1] unfortunately contained a mistake. All occurrences in the main text referring to the research carried out in the following countries; Brazil, El Salvador and Colombia should instead be replaced with “Brazil, Honduras and Panama”. The original version of this article has been updated to reflect this change.

## References

[1] Moazzam Ali, Rachel Folz, Kelsey Miller, Brooke Ronald Johnson Jr and James Kiarie. A study protocol for facility assessment and follow-up evaluations of the barriers to access, availability, utilization and readiness of contraception, abortion and postabortion services in Zika affected areas. 10.1186/s12978-017-0283-8. 10.1186/s12978-017-0283-8 PMC5288873 28153043

